# Peripheral T follicular helper cells from H1N1/09 vaccine nonresponders fail to induce antigen-specific antibody production in vitro

**DOI:** 10.1186/1742-4690-9-S2-P277

**Published:** 2012-09-13

**Authors:** A Parmigiani, S Pallikkuth, SY Silva, M Fischl, R Pahwa, S Pahwa

**Affiliations:** 1 University of Miami, Miami, FL, USA

## Background

Mechanisms underlying poor Ab responses to vaccines in well controlled HIV infected patients are not fully understood. In this study we have examined the role of a novel subset, the peripheral T follicular helper cells (pTFH) in Ab production.

## Methods

The study was conducted in cryopreserved cells of 16 HIV-infected, ART-treated individuals and 8 healthy donors (HD) who had been given a single dose of H1N1/09 influenza vaccine in 2009. Only 8 of the 16 patients and all HD had a flu-Ab response at 4 wks post vaccination. Vaccine responder (VR) and non responder (VNR) patients were equivalent in mean age, viral load, CD4 and CD8 T and B cells. B (CD20+) and TFH (CD3+ CD4+ CD45RA– CXCR5+) cells were purified by cell sorting on a FACS ARIA and co-cultured. IgG levels in culture supernatants were determined by ELISA. B and T cell phenotypic analysis was performed by multicolor flow cytometry. Differences between groups were analyzed by Student t-test or the 2-sample Wilcoxon rank-sum (Mann-Whitney) test.

## Results

Frequency of pTFH was equivalent in HIV+ patients and HD before vaccination. pTFH cells underwent significant expansion at wk4 compared to baseline in VR patients (p= 0.003) and HD (p= 0.001) with increased frequency of Ki67+ cells. In VR, H1N1-specific IgG production was evident in CD4+ CXCR5+/B cell co-cultures but not in CD4+ CXCR5–/B cell co-cultures [HIV+ n = 3; p = 0.043] and [HD n=3; p = 0.014], concurrently with increase in frequencies of plasmablasts. These changes were not seen in vaccine nonresponders.

## Conclusion

pTFH cells promote antigen-specific Ab production by B cells in vitro. Although their relationship to lymph node germinal center TFH has not been clarified, analysis of pTFH function represents a novel and easily accessible surrogate marker for vaccine responsiveness in stable HIV infected patients with equivalent CD4 T cells.

